# Nutritional vitamin D supplementation and health-related outcomes in hemodialysis patients: a protocol for a systematic review and meta-analysis

**DOI:** 10.1186/s13643-015-0002-x

**Published:** 2015-02-21

**Authors:** Anita Mehrotra, Wai-Yin Leung, Tannia Joson

**Affiliations:** Division of Nephrology, Department of Medicine, Icahn School of Medicine at Mount Sinai, One Gustave L. Levy Place, Box 1243, New York, NY 10029 USA

**Keywords:** Vitamin D, 25(OH)D, Ergocalciferol (D2), Cholecalciferol (D3), Hemodialysis, Clinical trial, Systematic review, Protocol

## Abstract

**Background:**

The prevalence of vitamin D deficiency in hemodialysis patients is high. While most hemodialysis patients are treated with activated vitamin D (1,25[OH]_2_D) to prevent renal osteodystrophy, clinical practices of the screening and treatment of 25(OH)_2_D deficiency are highly variable. It is unclear if nutritional vitamin D supplementation with D2 or D3 provides an additional clinical benefit beyond that provided by activated vitamin D treatment in this population.

**Methods/design:**

We will conduct a systematic review of nutritional vitamin D (D2/D3) supplementation and health-related outcomes in hemodialysis patients according to Preferred Reporting Items for Systematic Reviews and Meta-Analyses (PRISMA) guidelines. The primary objective is to assess the impact of nutritional vitamin D supplementation on clinical outcomes relevant in hemodialysis patients, such as mortality, cardiovascular events, infections, and fractures. Secondary outcomes will include anemia, hyperparathyroidism, medication use (erythrocyte-stimulating agents, activated vitamin D), and quality of life. We will search MEDLINE, Scopus, Web of Science, and ClinicalTrials.gov for randomized, controlled trials of nutritional vitamin D supplementation (ergocalciferol/D2 or cholecalciferol/D3) in chronic hemodialysis patients. The Cochrane Risk Assessment Tool will be used to assess the quality of eligible studies. We will perform meta-analyses using standard techniques for the outcomes listed above if pooling is deemed appropriate/sufficient. The results of this systematic review may highlight gaps in our knowledge of the relevance of nutritional vitamin D in end-stage renal disease, allowing for the informed design of clinical trials assessing the impact of nutritional vitamin D therapy in the hemodialysis population in the future.

**Systematic review registration:**

PROSPERO CRD42014013931

## Background

Over 600,000 individuals live with end-stage renal disease (ESRD) in the United States [1]. Among ESRD patients on hemodialysis (HD) as a renal replacement therapy, cardiovascular disease is the leading cause of death, and large cohort studies have shown that non-traditional cardiovascular risk factors, such as vitamin D deficiency, are associated with increased mortality in this population [2-8]. Whether or not this risk is mitigated by treatment of vitamin D deficiency is unknown.

Vitamin D is a steroid hormone, traditionally known for its role in the regulation of calcium, phosphorus, and bone metabolism. It can be derived from dietary sources (D2 and D3) and/or produced in the skin by exposure to UV light (D3). It is 25-hydroxylated in the liver and then undergoes 1-alpha-hydroxylation to its active form, 1,25-dihydroxyvitamin D [1,25(OH)_2_D], by 1-alpha-hydroxylase (CYP27B1) in the kidney (in the context of normal renal function) as well as in extra-renal sites. 1,25(OH)_2_D binds to the intracellular vitamin D receptor (VDR) to alter gene transcriptional profiles and mediate downstream effects [9].

Vitamin D deficiency is widespread among the general population [10] and is highly prevalent in patients with ESRD on hemodialysis. The concentration of 25(OH)D is the main clinical indicator of vitamin D status and is used to define vitamin D deficiency [11]. According to the Institute of Medicine (IOM), there is an elevation in the risk of experiencing adverse health consequences of vitamin D deficiency at 25(OH)D concentrations <20 ng/mL [12]. Multiple reports from various centers worldwide have documented that over 80% of HD patients have serum vitamin D levels within the insufficient/deficient range [13,14]. The etiologies of hypovitaminosis D in the ESRD population are not clear but include limited sunlight exposure, reduced UVB-induced vitamin D synthesis in the skin, and disturbed vitamin D metabolism [15-18]. Because renal CYPR27B1 is essentially absent in patients with ESRD, the levels of circulating 1,25(OH)_2_D are also commonly well below the normal range, contributing to secondary hyperparathyroidism and bone loss. While many of these ESRD patients are treated with 1,25(OH)_2_D (or its equivalent) during hemodialysis for the prevention of uremic bone disease, this alone may not fully satisfy the vitamin D requirement. Increasing experimental evidence indicates that many vitamin D target tissues, including intestinal and immune cells, express CYP27B1 and the VDR [19-21]. Recent data suggest that *local* production of 1,25(OH)_2_D is required to obtain maximal calcium absorption, expression of antimicrobial peptides, and anti-inflammatory effects [19-25]. Because 25(OH)D is the immediate precursor of 1,25(OH)_2_D, low serum 25(OH)D concentrations are thus hypothesized to prevent the production of 1,25(OH)_2_D in target tissues, potentially promoting inflammation and reducing other vitamin D effects.

Based on published data demonstrating the prevalence of vitamin D deficiency in hemodialysis patients and the possible extra-skeletal benefits of nutritional vitamin D supplementation, it is imperative to establish recommendations to guide clinical practice and research in this arena. The purpose of the proposed systematic review is to provide the best available evidence on the treatment of vitamin D deficiency in the hemodialysis population and, if the evidence is inadequate, to highlight areas where further original research is required.

## Methods/design

### Research objectives

We will conduct a systematic review to determine the clinical impact of nutritional vitamin D supplementation on outcomes in hemodialysis patients (concurrently treated with 1,25[OH]_2_D). The pre-specified primary outcomes will be mortality, cardiovascular events, infections, and fractures. The secondary outcomes will include anemia, hyperparathyroidism, medication use (erythrocyte-stimulating agents, activated vitamin D), and quality of life. These pre-defined outcomes may be adjusted during the review, depending on the outcomes identified in eligible studies.

### Types of studies

Only randomized, controlled trials (RCTs) of nutritional vitamin D (D2/ergocalciferol and/or D3/cholecalciferol) supplementation will be included in this systematic review. The choice to restrict the review to randomized controlled trials was made to eliminate the bias and confounding inherent in observational and uncontrolled studies of nutritional vitamin D repletion in this population. We may, however, find a paucity of eligible studies using this restriction strategy. If we are unable to find at least five eligible RCTs for the purposes of this systematic review, we will broaden our inclusion criteria (see below) to include non-randomized and/or uncontrolled interventional studies of nutritional vitamin D supplementation in HD patients using the Cochrane Effective Practice and Organization of Care (EPOC) approach to categorize the types of studies [26].

### Search strategy

We will employ a broad electronic search strategy, using MEDLINE, Scopus, and Web of Science to identify published abstracts and manuscripts of randomized controlled trials of nutritional vitamin D supplementation in hemodialysis patients. To identify unpublished studies and assess publication bias, we will also search ClinicalTrials.gov for registered clinical trials of nutritional vitamin D supplementation in hemodialysis patients. With the goal of prioritizing sensitivity while remaining specific to clinical trials, the search strategy will include the relevant key terms: ‘cholecalciferol’ or ‘D3’ or ‘ergocalciferol’ or ‘D2’ and ‘hemodialysis’ and ‘trial’. We will also review the references of included manuscripts to potentially identify any missed trials. The corresponding authors of all identified eligible studies (published and unpublished) will be contacted to provide de-identified data for the purposes of meta-analysis.

### Study screening and inclusion

All abstracts returned using the search strategy above will be screened by two independent investigators (WL and TJ) using the inclusion/exclusion criteria outlined below. In cases where an abstract is not available, the full text of the manuscript will be reviewed and evaluated instead. If the two independent investigators disagree on whether or not a particular study meets the inclusion criteria for the review, a third investigator (AM) will serve as a ‘tiebreaker’. The full text of all studies deemed eligible for inclusion will then be reviewed by all investigators for analysis.

### Inclusion criteria

We will include all randomized controlled trials of nutritional vitamin D (D2/ergocalciferol and/or D3/cholecalciferol) supplementation in adult and pediatric male and female chronic hemodialysis patients, regardless of outcome(s) measured. Only randomized, controlled interventional studies will be included. However, we will not restrict studies based on outcome(s) assessed, as we anticipate a limited number of true RCTs. We acknowledge that the type of nutritional supplementation (D2 vs D3), dose and preparation of supplement(s), and degree of vitamin D deficiency will vary among studies, but in order to maximize the total study population, we will not require any one particular supplement formulation/regimen or dosing schedule. Studies published in languages other than English will be translated and included if they otherwise meet all inclusion/exclusion criteria. As mentioned above, if we are unable to find at least five eligible RCTs for the purposes of this systematic review, we will broaden our inclusion criteria to include non-randomized and/or uncontrolled interventional studies of nutritional vitamin D supplementation in HD patients.

### Exclusion criteria

All case reports/series, review articles, observational studies, and non-randomized or uncontrolled interventional studies will be excluded. All animal studies will be excluded. We are also excluding studies of peritoneal dialysis (PD) and kidney transplant patients as these ESRD populations do not typically receive intravenous 1,25(OH)_2_D therapy, an important potential confounder.

### Data extraction

Data extraction will be performed in a standardized fashion for all trials included in the final review using a pre-formatted database. All extracted data will be verified by a second investigator to ensure accuracy and completeness. Study investigator information, study sites/locations, sample size, inclusion/exclusion criteria, type and dose of nutritional vitamin D supplements, follow-up time, and patient characteristics (age, gender, race, dialysis vintage, comorbidities, dialysis access, medications) will be collected as variables for analysis. Baseline and follow-up 25(OH)D concentrations will also be collected, regardless of time of testing. All outcome variables will also be collected, regardless of number of studies with that outcome assessed.

### Quality assessment

The internal validity of all eligible randomized controlled trials will be evaluated using the Cochrane Risk Assessment Tool. The tool evaluates studies based on seven criteria: 1) randomization generation, 2) allocation concealment, 3) blinding of outcome assessors, 4) blinding patients/study personnel, 5) incomplete outcome data (that is, lost to follow-up), 6) selective outcome reporting, and 7) other risks of bias. Other features to be incorporated in the quality assessment include sample size and statistical analysis (was it appropriate?). In the event that we include non-randomized and/or uncontrolled interventional studies of nutritional vitamin D supplementation in HD patients (see ‘Types of studies’ above), we will use the Cochrane Effective Practice and Organization of Care Group Draft Risk of Bias Tool to assess quality and risk of bias [26].

### Analysis plan

We will develop a Preferred Reporting Items for Systematic Reviews and Meta-Analyses (PRISMA) diagram based on the search strategy and eligibility assessment to show the flow of included and excluded studies [27]. We will describe all included studies in detail in the Results section of the manuscript. Study name/identification, patient population, and detailed design will be discussed, with a comparison of outcomes assessed by the various studies. The descriptive statistics from each trial will also be included and described in as much detail as is available from the parent trial(s).

We will pool treatment effect estimates where possible using standard statistical techniques and RevMan software. However, if combining data across studies is not feasible due to excessive heterogeneity of outcomes, we will use descriptive methods to present the results of all eligible studies. We did not specify the duration of therapy in our inclusion criteria in an effort to avoid being too restrictive, but we will attempt to account for differences in treatment duration during the data analysis. We will group both studies of ergocalciferol (D2) and cholecalciferol (D3) together initially, and we will then perform a subsequent sensitivity analysis for each treatment separately, as the bioavailability of these two agents may differ [28]. Depending on the heterogeneity of dosing schedules, we may also perform separate analyses for differing dosing regimens. Finally, as follow-up time may vary from trial to trial, we will include time as a covariate in our final data analysis.

## Discussion

The National Kidney Foundation (NKF) Kidney Disease Outcomes Quality Initiative (KDOQI) guidelines do not address the treatment of vitamin D deficiency in hemodialysis patients beyond a recommendation for treatment with activated vitamin D during hemodialysis for the prevention of renal osteodystrophy [29]. Perhaps this is due to a perception that treatment with activated vitamin D, or 1,25(OH)_2_D, bypasses any need for its precursor, 25(OH)D. However, emerging evidence indicates that vitamin D may play a role in human health that goes beyond bone and mineral metabolism [11]. As such, determining the optimal strategy for screening and repletion of vitamin D deficiency in the ESRD population is of vital importance and is the purpose of the proposed systematic review.

We foresee several potential limitations with this systematic review: heterogeneity of clinical outcomes, substandard quality of existing studies, and a scarcity of randomized controlled trials, which are the focus of this project. Therefore, we anticipate that we may have to broaden our inclusion criteria to include non-randomized/uncontrolled interventional trials, particularly if we find fewer than five eligible RCTs for this review. We acknowledge that defining inclusion ‘sufficiency’ as five or more studies is somewhat arbitrary. However, we wanted to establish *a priori* a point at which we will consider changing our inclusion strategy to maximize the utility of performing a systematic review on this topic. We also anticipate that we may not be able to combine eligible studies for a meta-analysis, but we will nonetheless present our findings using descriptive methods, if necessary. This study protocol has been designed prior to any knowledge of the study data or outcomes from existing published (and non-published) literature. Hence, we are limited in our capacity to predict the results. Our hope is that the dissemination of this protocol will allow us to obtain feedback and constructive criticism of the methods of our study before it is conducted.

In conclusion, the proposed systematic review will provide insight into the clinical impact of nutritional vitamin D repletion in hemodialysis patients concurrently receiving treatment with activated vitamin D. The results have the potential to inform national and international guidelines on the care and management of vitamin D deficiency in this population. The review will also help to highlight areas requiring further research on this topic.

## Abbreviations

ESRD: end-stage renal disease
HD: hemodialysis
IOM: Institute of Medicine
KDOQI: Kidney Disease Outcomes Quality Initiative
NKF: National Kidney Foundation
PRISMA: Preferred Reporting Items for Systematic Reviews and Meta-Analyses
RCT: randomized controlled trial
VDR: vitamin D receptor
