# Erratum to: Nutritional vitamin D supplementation and health-related outcomes in hemodialysis patients: a protocol for a systematic review and meta-analysis

**DOI:** 10.1186/s13643-015-0086-3

**Published:** 2015-08-11

**Authors:** Anita Mehrotra, Wai-Yin Leung, Tannia Joson

**Affiliations:** Division of Nephrology, Department of Medicine, Icahn School of Medicine at Mount Sinai, One Gustave L. Levy Place, Box 1243, New York, 10029 NY USA

## Erratum

Unfortunately, the original version of this article [1] published with an incorrect citation. The citation has now been updated to 4:93, which is the correct citation for the article. The Publisher apologizes for any inconvenience caused.
